# OCAM Regulates Embryonic Spinal Cord Stem Cell Proliferation by Modulating ErbB2 Receptor

**DOI:** 10.1371/journal.pone.0122337

**Published:** 2015-04-13

**Authors:** Loïc Deleyrolle, Jean-Charles Sabourin, Bernard Rothhut, Hiroko Fujita, Pierre-Olivier Guichet, Marisa Teigell, Chantal Ripoll, Norbert Chauvet, Florence Perrin, Daria Mamaeva, Tetsuo Noda, Kensaku Mori, Yoshihiro Yoshihara, Jean-Philippe Hugnot

**Affiliations:** 1 Department of Neurosurgery, College of Medicine, University of Florida Gainesville, Gainesville, Florida, United States of America; 2 INSERM U1051, Institute for Neuroscience, Hôpital Saint Eloi, Montpellier, France; 3 RIKEN Brain Science Institute, Saitama, Japan; 4 INSERM U661, Department of Endocrinology, Institute of Functional Genomics, Montpellier, France; 5 Department of Cell Biology, Cancer Institute, Japanese Foundation for Cancer Research, Tokyo, Japan; 6 Department of Physiology, Graduate School of Medicine, The University of Tokyo, Tokyo, Japan; 7 University of Montpellier 2, Montpellier, France; National Cancer Institute, UNITED STATES

## Abstract

The proliferation and differentiation of neural stem cells are tightly controlled by intrinsic and extrinsic cues. Cell adhesion molecules are increasingly recognized as regulators of these processes. Here we report the expression of the olfactory cell adhesion molecule (OCAM/NCAM2/RNCAM) during mouse spinal cord development and in neural stem cells cultured as neurospheres. OCAM is also weakly expressed in the dormant adult stem cell niche around the central canal and is overexpressed after spinal cord injury. Both transmembrane (TM) and glycosylphosphatidylinositol (GPI)-linked isoforms are present in neurospheres. Electron microscopy and internalisation experiments revealed a dynamic trafficking of OCAM between the membrane and intracellular compartments. After differentiation, OCAM remains in neurons and oligodendrocytes whereas no expression is detected in astrocytes. Using OCAM knockout (KO) mice, we found that mutant spinal cord stem cells showed an increased proliferation and self-renewal rates although no effect on differentiation was observed. This effect was reversed by lentivirus-mediated re-introduction of OCAM. Mechanistically, we identified the ErbB2/Neu/HER2 protein as being implicated in the enhanced proliferation of mutant cells. ErbB2 protein expression and phosphorylation level were significantly increased in KO cells whereas no difference was observed at the mRNA level. Overexpression of ErbB2 in wild-type and mutant cells also increased their growth while reintroduction of OCAM in mutant cells reduced the level of phosphorylated ErbB2. These results indicate that OCAM exerts a posttranscriptional control on the ErbB2 signalling in spinal cord stem cells. This study adds further support for considering cell adhesion molecules as regulators of the ErbB signalling.

## Introduction

Glial and neuronal cells of the central nervous system (CNS) originate from multipotential cells, called neural stem cells (NSC) residing in the neuroepithelium. The production of differentiated cells at the correct time and location is tightly controlled by complex cellular mechanisms such as asymmetric division and diffusible molecules including morphogens and cytokines. NSC also persist in the adult CNS in specific locations called niches, mainly the subventricular zone and the hippocampus, which provide an appropriate environment to maintain the cells in an immature state. These NSC underlie adult neurogenesis, which is tightly regulated by diverse environment cues as well as by physical exercise. NSC are also present around the central canal of the adult spinal cord although no associated neurogenesis is observed [1,2]. Several models have been designed to study the properties of NSC in vitro and the propagation of these cells as free-floating neurospheres is now commonly used [3]. This has enabled the discovery of key genes and molecules regulating the differentiation and self-renewal of these cells. Along this line, an important role for cell adhesion molecules (CAMs) has been recognized [4]. These proteins are no longer considered as plain cell-cell glue but in contrast as critical proteins linking the cellular environment with the molecular pathways regulating cell proliferation, differentiation and survival. CAMs modulate intracellular signal transductions through interactions mediated by their cytoplasmic domains or through interactions between their extracellular part and growth factor receptors. A role for CAMs of the immunoglobulin (Ig) superfamily (mostly L1CAM and NCAM1) [5–7] and cadherins [8–10] has been described in the control of proliferation and differentiation of embryonic and adult neural stem cells.

Whereas much is known on neurosphere-cultured NSC derived from the embryonic and adult brain, much less is known on spinal cord neurospheres. These cells display distinct properties from brain-derived neurospheres and maintain the expression of spinal cord developmental genes such as Hox genes [11]. In a previous study, we showed that the self-renewal and differentiation of these cells is controlled by endogenous and exogenous cytokines [12]. In this new study, we focused on a specific and scarcely studied member of the CAM family, namely the olfactory cell adhesion molecule OCAM (also called NCAM2 or RNCAM) [13]. We identified the expression of OCAM in embryonic and adult spinal cord neurosphere cells and studied its role in this context. OCAM belongs to the Ig superfamily and its expression and role have been mainly investigated in the olfactory system where it is expressed by subpopulations of sensory neurons in the olfactory epithelium, which project to restricted spatial zones in the olfactory bulb [13]. This intriguing expression pattern has made OCAM the leading candidate as determinant of broad rhinotopy between olfactory epithelium and olfactory bulb. However, both knockout experiment [14] and ectopic expression of OCAM [15] have not validated this hypothesis. Instead, OCAM was found to have a role in the compartmental organization of dendrites and axons within the olfactory glomeruli [14] as well as a function in the accurate selection of olfactory sensory neurons target. At the functional level, OCAM mutant mice displayed a reduction in mitral cell synchronous activity in olfactory glomeruli [16] and also showed an increased olfactory acuity [14]. Like NCAM1, OCAM proteins can make homophilic interactions to mediate cell-cell adhesion properties. However it is not glycosylated with polysialic acid [13], a posttranslational modification known to be a major regulator of NCAM1-mediated processes. The heterophilic interaction of OCAM with the PrPc prion protein has also been reported but the biological significance of this interaction remains indefinite [17].

Here we report a previously undocumented role for OCAM in spinal cord stem cells. This cell adhesion molecule was found to exert a role on the self-renewal and proliferation of these cells. Mechanistically, we uncovered a functional link between OCAM and the ErbB2/Neu/HER2 protein.

## Materials and Methods

### Animals

Mice were handled following the guidelines of the Animal Care and Use Committee of the Institut National de la Santé et de la Recherche Médicale (INSERM) who approved this study in accordance with the European Council directive (2010/63/UE) for the protection and use of vertebrate animals. Every effort was made to minimize the number and suffering of animals. Wild-type (C57BL6/J, Charles River, France) and OCAM-KO mice were housed in the animal facility under closely controlled environmental conditions (12-hour light/dark cycle, room temperature 22°C), and fed ad libidum (food and water). For neurosphere cultures, adult or embryonic spinal cords were dissected from mice euthanatized by intraperitoneal injection of sodium pentobarbital (100 mg/kg).

For spinal cord injury, adult Swiss mice (12 weeks) were anesthetized using isoflurane gas (1.5%). T9–T10 laminectomy was performed to expose the spinal cord. Four needle penetrations (30G) were done (staggered holes, 2 on each side of the posterior spinal vein). Penetration depth was approximately 1 mm. Muscles and skin were sutured and animal placed on heated pads until they wake-up. Mice were euthanatized 72h after injury by intraperitoneal injection of sodium pentobarbital (100 mg/kg).

### Generation of *OCAM*-deficient mice

OCAM-deficient mice were generated with a standard gene-targeting method [18]. An 18-kb DNA fragment containing the exon 1 of OCAM was isolated from 129/Sv mouse genomic DNA library. The targeting vector was designed to delete 153-bp sequence covering the 3’-end of exon 1 and intron 1 by replacement with tau-lacZ [19] and a loxP-neo cassette [20], which disrupts the initiation methionine and signal peptide sequence of OCAM gene. 8-kb upstream and 1.4-kb downstream arms were used as homologous flanking sequences. Diphtheria toxin A subunit gene (pMC1-DT-A, [21]) was introduced as a negative selection marker against random integration of the vector. Electroporation of the targeting vector into J1 ES cells followed by G418 selection yielded three positive homologous recombinants as determined by Southern blot analysis with 5’ probe (a 0.6-kb Xba I/Hae III fragment derived from upstream of *Ocam* exon 1), neo probe (a 0.6-kb Pst I fragment of neo) and 3’ probe (a 0.5-kb Xba I/Sac I fragment derived from *Ocam* intron 1). Chimeric C57BL/6 males that transmitted the mutant allele were obtained and backcrossed with C57BL/6 mice. A *loxP-neo* cassette was removed by mating with CAG-Cre female mice [22]. For genotyping PCR, the following primers were used: a wild-type allele forward primer (5’-TCTCCGGGGCTGGACTTAATAACTTTGG-3’ or a mutant allele forward primer (5’-ATCATGTCTGGATCGGGCGAGCTCG-3’) paired with a common reverse primer (5’-TCAAGAACCACCGCAACTTTGGTGC-3’).

### Cell culture

Neurosphere cultures were derived from E13-E14 embryonic and adult mouse spinal cord as previously described in [11,12] and grown in defined media supplemented with EGF/FGF2. Cell growth was measured by seeding dissociated cells (1000 or 5000 cells per well) in 1 ml of media in 24-well plates coated with poly-HEMA (Sigma) to inhibit cell adherence. After 5 or 7 days, the neurospheres were directly dissociated by addition of trypsin in the wells (0.5% final) and the cell number was measured with an automated cell counter (Z2, Beckman Coulter). To determine the neurosphere forming unit (Nsfu), the neurospheres were enzymatically dissociated and clonally seeded at 1 cell/well in 96-well plates using an automatic cell seeding device (Aria cytometer BD). After two weeks, the number of spheres with a diameter >500μm was visually determined. Nsfu = number of spheres/96 wells x 100. Neurosphere differentiation was obtained by culturing them for 4 days without growth factor on poly-D-lysine coated coverslips. Neurosphere transfection was performed using an Amaxa apparatus with a neural stem cell tranfection kit (Amaxa). ErbB2 (Addgene n°10917) and control GFP plasmids were obtained from Addgene and amplified using endotoxin-free kits (Qiagen). Purified recombinant OCAM coupled to Fc fragment (OCAM-Fc) and control Fc fragment, produced in CHO cells, were purchased from R&D.

### Lentivirus

OCAM-TM and OCAM-GPI cDNAs [13] were subcloned in BamH1-Xho1 sites of pHRTK plasmid (gift from Dr P. Corbeau, IGH, Montpellier, France) and lentiviruses were produced, concentrated and titrated (p24) as described in [23]. For infection, 100 ng of p24 was used for 10^5^ cells.

### Cytometry

Mechanically-dissociated neurospheres were incubated in 1% BSA-PBS buffer containing 1μg/10^6^ cells of goat anti-OCAM antibody (R&D #AF778) or control IgG antibody for 30 min on ice. Cells were washed in 10 volumes of buffer, centrifuged and incubated with Alexa488-conjugated donkey anti-goat IgG antibody for 30 min. Cells were washed again, resuspended in PBS buffer containing 1 μg/ml of propidium iodide and immediately analysed on an FACSAria cytometer (BD).

### Electron microscopy of OCAM

Neurospheres were fixed with 4% paraformaldehyde and 0.2% glutaraldehyde in phosphate buffer 0.1M pH 7.3 during 30 min at room temperature, rinsed in phosphate buffer (3x10 min) and incubated with 0.3% hydrogen peroxide to eliminate endogenous peroxydase activity. Neurospheres were incubated overnight with anti-OCAM antibody [13] diluted 1:500 in phosphate buffer containing serum 5% and saponin 0.05%. Biotinylated anti-rabbit secondary antibody and Vectastain Elite ABC Kit were used according to manufacturer’s recommendations to revealed OCAM protein. Neurospheres were post-fixed in osmium tetroxide 0.5% in phosphate buffer for 1h at 4°C, dehydrated in ethanol (30% to 100%) and propylene oxide and then embedded in epoxy resin (Epon). Ultrathin 90nm sections were performed using an ultramicrotome (Leica). Sections were observed using a Hitachi 7100 transmission electron microscope.

### Immunofluorescence (IF)

Paraformaldehyde-fixed (4%, 20 min) neurospheres were processed for immunofluorescence as described in [12]. Antibody references and dilutions are: mouse-III tubulin (Sigma #T8660, 1:200), rabbit GFAP (Dako, #Z0334, 1:5000), mouse O4 (hydridoma supernatant, ¼), rabbit OCAM ([13]; 1:500), goat OCAM (R&D, #AF778, 1:200), rat OCAM (R&D, #MAB778, 1:2000), mouse Ki67 (BD, #556003, 1:500), goat Sox2 (Santa-Cruz, #sc-17320, 1:200), mouse Map2 (Sigma, clone AP-20, 1:500). Nuclei (blue on IF images) were stained with DAPI. Apoptosis was detected on coverslips by using the terminal deoxynucleotidyltransferase–mediated dUTP-biotin nick-end labeling (TUNEL) method with the ApopTag fluorescein in situ apoptosis detection kit (Chemicon) according to the manufacturer’s instructions.

To detect OCAM protein during spinal cord development, E10.5, E11.5 and E13.5 embryos (plug day = E0.5) were collected and immediately fixed for 1 hour in 4% paraformaldehyde. Embryos were cryopreserved in 30% sucrose, embedded in OCT, flash-frozen in liquid nitrogen with isopentane and cut with cryostat (13 μm). To detect OCAM in the adult dormant stem cell niche around the central canal, sections were prepared as described in [11].

### Internalisation assay

Growing embryonic spinal cord neurospheres were harvested, dissociated as single cells and then incubated during 30 minutes with an antibody recognizing OCAM extracellular epitopes (R&D, goat, #AF778, 1:50). Incubation was performed at 37°C (enabling protein trafficking) or 4°C (preventing or delaying OCAM internalization). Cells were fixed (4% paraformaldehyde, 20 min) and incubated with a green fluorescence-conjugated anti-goat secondary antibody (Jackson Immunoresearch Laboratories, 1:1000). The cells were then permeabilized (Triton X100, 0.1%, 10 min) and incubated with a red fluorescence-conjugated anti-goat secondary antibody (Jackson Immunoresearch Laboratories, 1:1000). Immunocomplexes were visualized using fluorescence microscope (Leica DMR) or laser confocal scanning microscope (Bio-Rad MRC 1024).

### Protein analysis

Protein extraction and Western blot (WB) were performed according to classical procedures. To detect OCAM in the medium, 10 ml of neurosphere-conditioned supernatant was syringe-filtered (10 μm), precipitated with 4 vol. of cold acetone (-20°C) and resuspended in 200 μl Laemmli loading buffer. Antibody references and dilutions are: rabbit OCAM ([13]; 1:500), goat OCAM (R&D, #AF778, 1:2000), ErbB2 (Cell Signaling, #2165, 1:1000), phospho-ErbB2 (Cell Signaling, #2241, 1:1000). To detect phosphorylated receptors in neurospheres, we used a mouse phospho-Receptor Tyrosine Kinase array (phospho-RTK array R&D, #ARY014) which was probed with OCAM KO and OCAM WT neurosphere extracts according to manufacturer’s recommendations. WB and arrays were quantified using ImageJ software.

### Laser-capture-microdissection and microarray screening

Thoracic parts of the spinal cord (T9–T10) of injured and control mice were rapidly removed, embedded in Tissue-Tek OCT Compound (Zoeterwoude, the Netherlands) and then frozen in SnapFrost Systeme (Excilone, France). Thick sections (30 μm) were cut and placed on polyethylene naphthalate (PEN) Membrane Frame slides (Carl Zeiss, Munich, Germany) under RNAase-free conditions. Specimens were briefly stained in 1% cresyl violet solution and laser-capture-microdissection was performed using a PALM MicroBeam microdissection system version 4.8 equipped with a P.A.L.M. RoboSoftware (P.A.L.M. Microlaser Technologies AG, Bernried, Germany). Total RNA was extracted from microdissected tissues using the ReliaPrep RNA Cell Miniprep System (Madison, WI USA) and RNA integrity was determined using RNA 6000 Pico Kit and Bioanalyzer (Agilent Technologies, Santa Clara, CA, USA).

### cRNA preparation, microarray hybridization and analysis

Hybridization targets were obtained following a double amplification procedure according to the protocol developed by Affymetrix (GeneChip Two-Cycle Eukaryotic Target Labeling Assay; Affymetrix, Santa Clara, CA, USA). A hybridization mixture containing 12 μg of biotinylated cRNA was generated and hybridized to Affymetrix HG-U133 Plus PM Array Strip (4 injured and 4 control animals). Chips were scanned using an Affymetrix GeneAtlas scanner and data files were generated with Affymetrix Expression Console v1.2.1.

The Transcriptome Analysis Console (TAC) 2.0 software was used for analysis. The full results obtained from this transcriptomics analysis of the adult spinal cord niche will be reported elsewhere.

### PCR

Total RNA was extracted from neurospheres (Qiagen) and reverse-transcribed (Superscript II, Promega) with random hexamers. Semi-quantitative PCR was performed with 100 ng of cDNA with Taq polymerase (Qiagen) for 30 cycles before gel electrophoresis in the presence of Sybr DNA dye. Quantitative PCR were performed using a Sybr PCR kit (Qiagen) and a LightCycler 480 apparatus (Roche). Primers sequences: OCAM-GPI (CCAAGCAGTGGCAAGAGTTT & GCATTCAGATGCCATGACTG); OCAM-TM (ATGGGCTACGAAGTGCAAAT & TGGACTCCCATCTTCATGGT); OCAM for QPCR (GGTGTCCCCTCAAGAGTTCA & GGATGGTGGTGACTTCCTCA); KI67 (GACTAGAAACCAAGCTGCGG & GCTGAGTTAAAGAGAGCCGC); ErbB2 (GAGCCTTCGGCACTGTCTAC & ACGTGGTTGGGACTCTTGAC); β-actin (AGACTTCGAGCAGGAGATGG & GTGCTAGGAGCCAGAGCAGT); GAPDH (TGTCCGTCGTGGATCTGAC & CCTGCTTCACCACCTTCTTG).

### Statistical analysis

All experiments were performed at least two times and most of them were done three times. Data are represented as means ± standard error of mean (SEM). Statistical differences in experiments were analysed using the Mann-Whitney rank sum test (GraphPad Prism software). Significances: *** (p<0.001), ** (p<0.01), * (p≤0.05).

## Results

### Strong expression of OCAM in cultured neural stem cells

**Fig 1:**
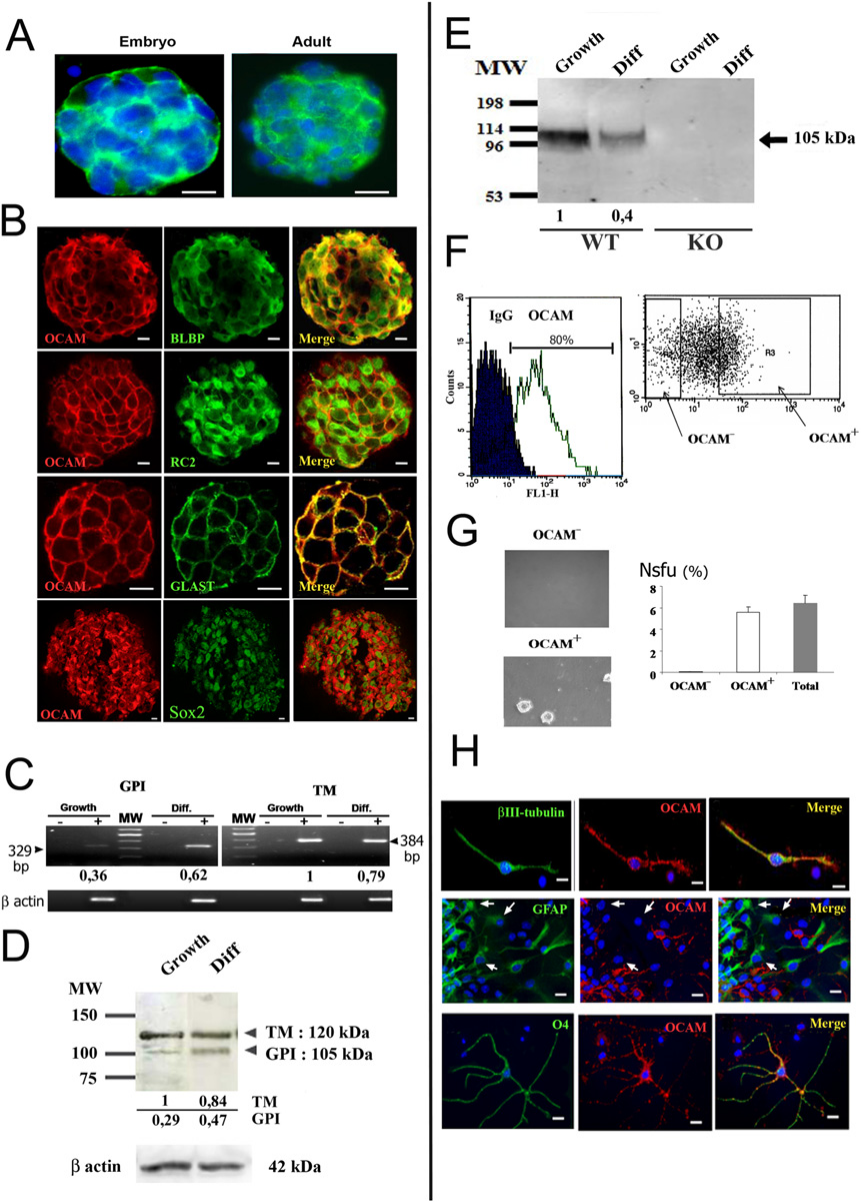
Expression of OCAM in spinal cord neural stem cells. (***A***): Detection by immunofluorescence of OCAM (green) in embryonic (left) and adult (right) spinal cord neurospheres. (***B***): OCAM is expressed by cells coexpressing BLBP, RC2, Sox2 and GLAST detected by immunofluorescence in embryonic spinal cord neurosphere cells (confocal images). A goat anti-OCAM antibody recognizing the external part of the protein was used for colabelling with BLBP, RC2 and GLAST whereas a rabbit antibody directed to the internal part of OCAM was used for labelling with anti Sox2 antibody. (***C***): PCR analysis of OCAM mRNA isoform expression in embryonic spinal cord neurospheres in growing condition (growth) or after 4 days of differentiation (diff.) Sizes (base pairs, bp) of amplified fragments are indicated. β-actin transcript amplification was used as an internal control. PCRs performed on RNA in which the reverse transcriptase step was omitted (-) demonstrate that the observed bands correspond to cDNA amplification and not genomic DNA. The TM isoform is predominant in growing neurospheres however only the GPI form increased after differentiation. (***D***): WB analysis of OCAM expression in protein extracts of embryonic spinal cord neurospheres in growing condition or after 4 days of differentiation. Both isoforms are detected. β-actin was used as an internal control. For C and D, the relative quantifications of bands are indicated at the bottom with TM band in growing condition taken as reference. (***E***): WB detection of OCAM in the media conditioned for 5–7 days by neurospheres. Media conditioned by OCAM KO cells was used as negative control. Compared to neurosphere extracts in (***D***), a small soluble form of OCAM, corresponding to the size of the GPI-linked isoform, is found in the supernatant. Relative quantifications of bands are indicated at the bottom. (***F***): Cytometry for OCAM. *Left*: Cytometric analysis indicated that the vast majority of neurosphere cells expressed OCAM. Incubation of the cells with non immune goat IgG was used as a control (IgG). *Right*: dot plot of OCAM-antibody incubated cells showing the population of OCAM^-^ and OCAM^+^ cells used to perform the neurosphere forming assays in (*G*). (***G***): Neurosphere-forming assays performed in clonal conditions showed that OCAM^-^ cells were unable to form neurospheres unlike OCAM^+^ cells which form neurospheres at the same rate as the total population. This latter went through the cytometer but was unsorted. The neurosphere forming cell unit (Nsfu) indicates the percentage of neurospheres >500μm obtained after clonal seeding of indicated cells. Photographs show cultures obtained from OCAM^-^ or OCAM^+^ cells one month after sorting. (***H***): Expression of OCAM after neurosphere differentiation. OCAM is expressed in neurons (βIII tubulin), oligodendrocytes (O4) but not in astrocytes (GFAP) (white arrows). Scale bars = 10 μm.

The expression of OCAM in neural stem cell cultures was initially uncovered using a gene array-based screen for cytokines and adhesion proteins present in E13-derived embryonic spinal cord neurospheres [12]. VCAM1 and OCAM adhesion molecules were found highly expressed in these cells (S1 Fig). The presence of OCAM protein was confirmed by immunofluorescence on neurospheres with a low passage number (3–4 times). OCAM was readily detected at the surface of most cells (Fig 1A). BLBP, RC2 and Sox2 are typical markers for neural stem cells in cultures [24] and OCAM co-stained with these proteins (Fig 1B). OCAM^+^ cells were also colabelled with GLAST, a marker for astrocytes but which is also expressed by neural stem cells [25,26]. The presence of OCAM was also detected during mouse spinal cord development (S2A–S2D Fig). Whereas it was barely detectable at E10.5, it was readily expressed at E11.5 in the marginal zone. At E13.5, OCAM became strongly expressed in the mantle zone and more moderately in the ependymal zone (S2A and S2B Fig). Co-labelling with neuronal (Map2) and stem cell (Sox2) markers indicated that OCAM was expressed by subpopulations of neuronal and ependymal cells (S2C and S2D Fig).

In adult, the spinal cord harbours a population of Sox2^+^ cells around the central canal. These cells constitute a neural stem cell niche from which neurospheres can be derived [1]. We thus examined the presence of OCAM in this niche as well as in neurospheres derived from this region. Whereas OCAM was strongly expressed in adult neurospheres (Fig 1A), it was weakly expressed by adult ependymal cells in vivo (S2E Fig). In adult, this niche is in a dormant state but is readily reactivated upon spinal cord injury [1]. We questioned the expression of OCAM in this situation and a clear increase of the protein was observed in ependymal cells 72h after injury (S2E Fig). This was confirmed at the RNA level using laser-microdissected central canal cells. Both microarray and QPCR showed that OCAM mRNA was increased approximately 3 time after injury (S2F Fig). A GPI-linked (GPI) and a transmembrane (TM) form of OCAM have been described in the olfactory epithelium [13]. We explored the presence of these isoforms by Western blot analysis and RT-PCR in embryonic neurosphere cultures. Both forms were present (Fig 1C and 1D) however OCAM-TM was predominant. In addition to cell surface expression, adhesion molecules have also been reported to be secreted by several cell types [27]. This incited us to analyse the presence of OCAM in a neurosphere culture medium that had been conditioned for 3 days. Indeed, a small soluble form of OCAM, with a size corresponding to the GPI-linked form, was predominantly detected in the supernatant (Fig 1E).

Neurosphere cultures are heterogeneous and contain few bona-fide neural stem cells able to form large and long-term passageable neurospheres when seeded at clonal or low-density plating. These neurosphere-forming cells (typically 1–10% of the neurosphere culture) are mixed with progenitors showing a more limited capacity for proliferation and new neurosphere formation [3]. No good marker currently exists to identify these neurosphere-forming cells prospectively in the neurosphere cultures. To examine whether OCAM was indeed expressed by this subpopulation of neurosphere-forming cells, we performed cell-sorting experiments using an antibody directed against the external part of OCAM. Approximately 80% of the neurosphere cells expressed OCAM (Fig 1F) and we assessed the neurosphere-forming capacity of OCAM^+^ and OCAM^-^ sorted cells by plating them at clonal density. Unsorted cells were used as a control. After two weeks, neurosphere counting indicated that approximately 6% of OCAM^+^ cells had formed new neurospheres, which is equivalent to the rate obtained with unsorted cells (Fig 1G). These neurospheres could be subcultured for at least 7 passages. This demonstrated that in neurosphere cultures, the subpopulation of neurosphere-forming cells expressed OCAM. In contrast OCAM^-^ cells lacked the ability to generate neurospheres (Fig 1G).

Finally, we explored the expression of OCAM when neurospheres were differentiated into astrocytes, oligodendrocytes and neurons. These cells can be identified respectively by the expression of GFAP, O4 and β-III tubulin markers and the expression of OCAM was examined by co-labelling. As shown on Fig 1H, after 4 days of differentiation, OCAM was found in neuronal and oligodendrocytic cells whereas it was not or barely expressed in astrocytic cells. WB and PCR analyses revealed that both OCAM isoforms were still expressed after differentiation, however the GPI form appeared to be upregulated relatively to the TM form (Fig 1C and 1D). The soluble OCAM was also detected in the media in this condition (Fig 1E).

### Neural stem cells internalise OCAM

**Fig 2:**
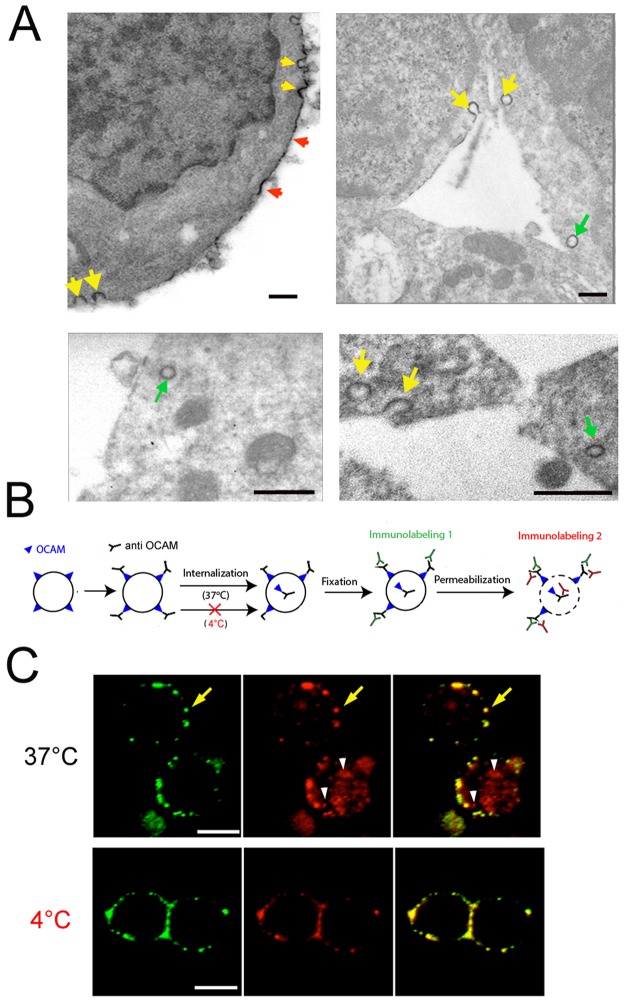
OCAM subcellular localization and internalization. (***A***): Electron microscopy of OCAM. Microphotographs of peroxydase-immunodetected OCAM shows its association with the cytoplasmic membrane (red arrows), with the membrane undergoing endo- or exocytosis processes (omega-like structures, yellow arrows) and with intracellular vesicles (green arrows). Scale bars = 0.5μm. ***(B)***: Schematic of the internalization assay. (***C***): OCAM membrane staining (yellow arrows) is observed in both conditions (37°C, upper panels and 4°C, lower panels). Intracellular labelling of OCAM (white arrowheads), revealed by membrane permeabilization, is only observed when cells are incubated at 37°C with the OCAM antibody. Scale bars = 10 μm.

Electron microscopy was performed to more precisely localize OCAM in neurosphere cells. This confirmed the membranous expression of this protein (red arrows, Fig 2A) but also revealed the existence of OCAM-coated vesicles in the cytoplasm (green arrows Fig 2A). In addition, we observed images of OCAM-positive vesicles undergoing endocytosis or exocytosis at the membrane (yellow arrows, Fig 2A). These images prompted us to explore whether OCAM could be internalized upon binding. To test this hypothesis, we performed a functional assay (Fig 2B) where we incubated living cells at 37°C with a primary anti-OCAM antibody recognising the extracellular part of the protein and after 30 min, the cells were fixed. Without cellular permeabilization, a green fluorescence-conjugated secondary antibody recognized OCAM at the cell surface as expected (Fig 2C, yellow arrows). However following cellular permeabilization with detergent, incubation with a red fluorescence-conjugated secondary antibody additionally revealed in the same cell the presence of an intracellular pool of OCAM which was internalized during the incubation time (Fig 2C, white arrowheads). The same experiment performed at 4°C, showed the absence of the intracellular pool of OCAM thus demonstrating that its internalisation is a temperature-dependent active process (Fig 2C).

### Loss of OCAM affects neural stem proliferation and self-renewal

**Fig 3:**
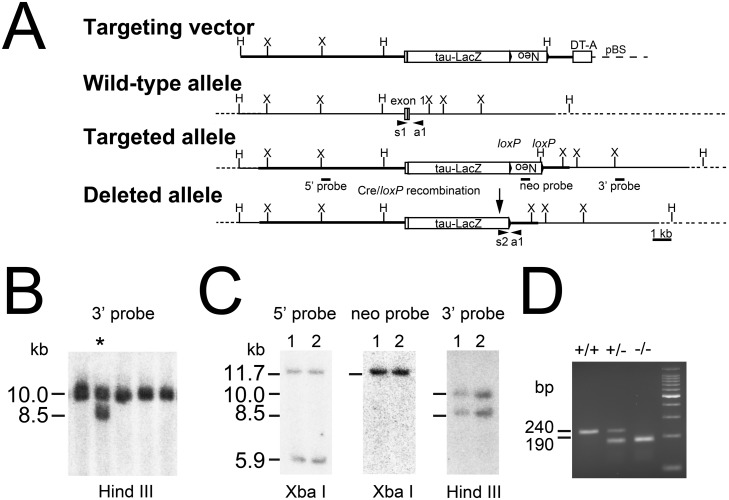
Generation of OCAM-deficient mice. (***A***): Gene targeting strategy to generate *OCAM*-deficient mice. The *pgk-neo* gene (Neo) flanked by loxP site and *tau-lac Z* gene (tau-LacZ) was inserted into the first exon of OCAM gene. H, Hind III; X, Xba I; pBS, pBluescript II. (***B***): Southern blot analysis of ES clones. ES cell DNA was digested with Hind III and hybridized with OCAM-3' probe indicated in (***A***). The homologous recombinant clone is indicated with asterisk (*). (***C***): The correct integration of the targeting vector was confirmed by Southern blotting of Hind III or Xba I digest with probes indicated in (A) on two of positive clones. (*D*): PCR genotyping of WT (wild-type, +/+), heterozygous (+/-), and homozygous KO (knock out,-/-) OCAM mutant mice. The primer specific to each allele (s1–s2, a1) were indicated.

**Fig 4:**
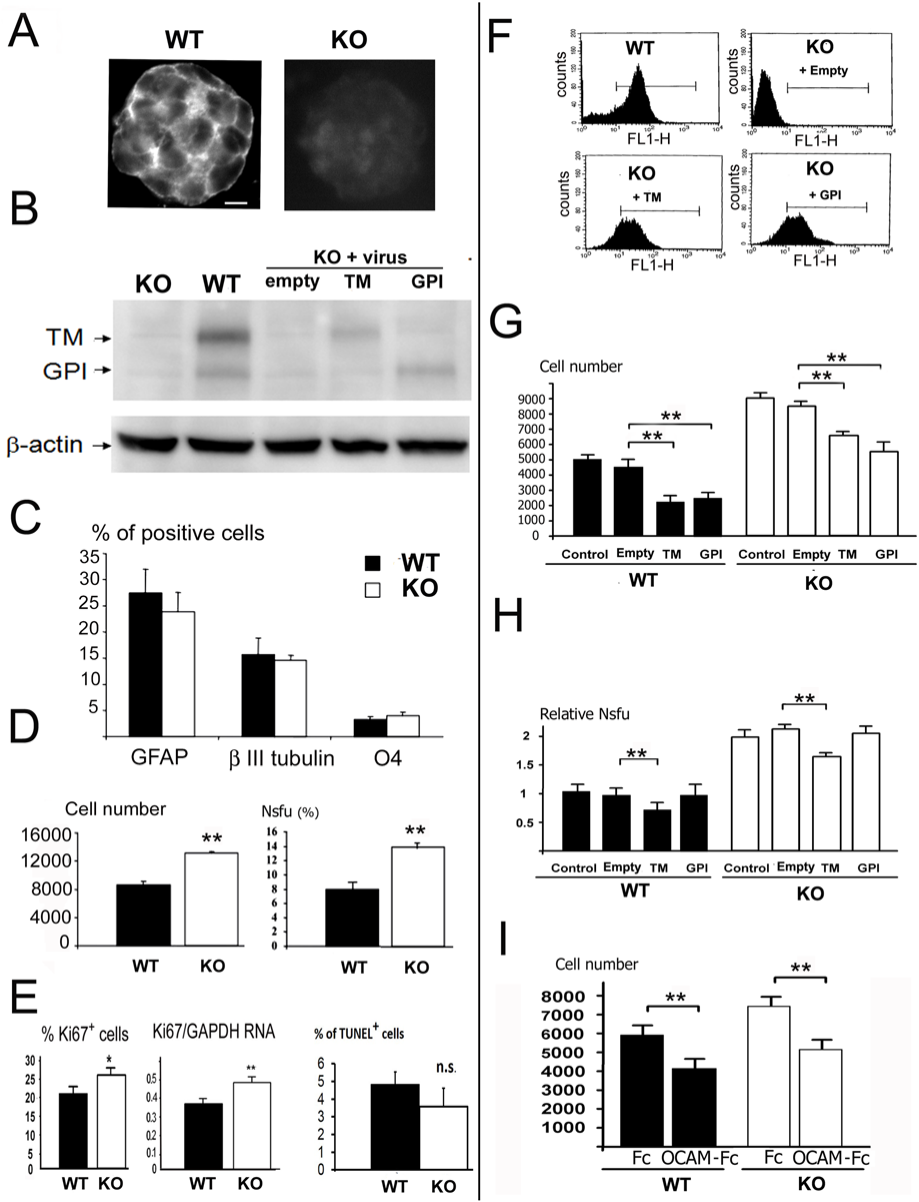
Properties of OCAM KO neurospheres. (***A***): Immunofluorescence detection of OCAM in KO and WT embryonic spinal cord neurospheres. Scale bar = 10 μm. (***B***): Western blot analysis of OCAM in indicated protein extracts. Vector, TM and GPI indicates OCAM KO neurospheres which were infected with respectively empty, OCAM-TM cDNA and OCAM-GPI cDNA lentiviruses. β-actin was used as an internal control. (***C***): Differentiation of KO and WT cultures. The % of astrocytic, neuronal and oligodendrocytic cells detected by the indicated markers are indicated. No significant difference was observed (n = 10 fields). (***D***): Growth properties of KO neurospheres. *Left*: Cell numbers obtained 7 days after seeding of indicated cultures (n = 7 wells). *Right*: neurosphere forming cell unit (Nsfu) of indicated cultures (n = 4). (***E***): *Left*: percentage of Ki67^+^ cells in KO and WT cultures (n = 6). *Middle*: QPCR quantification of Ki67/GAPDH mRNA (n = 4). *Right*: % of apoptotic cells detected by TUNEL assay. n.s. = not significant. (n = 4). (***F***): Cytometric analysis of OCAM expression in indicated cultures. Vector, TM and GPI indicates KO neurosphere cells which were transduced with respectively empty, OCAM-TM cDNA and OCAM-GPI cDNA lentiviruses. (***G***): Growth analysis of WT and KO neurospheres transduced by indicated lentiviruses. Cell numbers were measured 7 days after seeding (n = 7 wells). (***H***): Neurosphere forming assays of WT and KO neurospheres transduced by the indicated lentiviruses. Only OCAM-TM lentivirus decreased the Nsfu in both cultures (n = 4). Values represent relative Nsfu using control infected cells as the reference. (***I***): Effect of recombinant OCAM protein on cell growth. Cell numbers were measured after 7 days of growth of KO and WT cells in the presence of 7 μg/ml of OCAM-Fc protein or Fc fragment (n = 7).

The role of OCAM in neural stem cells was investigated through a loss-of-function approach using knockout (KO) mice. Fig 3 shows the generation of the OCAM-deficient mice. A null OCAM gene was created by inserting tau-lacZ and a *loxP-neo* cassette into exon 1 to disrupt the initiation methionine and signal peptide sequence of OCAM gene. No prominent abnormalities in spinal cord development were observed in mutant animals (data not shown). Neurosphere cultures were derived from spinal cords of OCAM KO embryos and the absence of the protein was confirmed by immunofluorescence labeling (Fig 4A) and Western blot analysis (Fig 4B). We first analysed whether the loss of OCAM could affect the differentiation of neurospheres however no significant differences could be measured in the formation of the different cell types between KO and WT cells (Fig 4C). We then compared the proliferation rate and clonogenic frequency between KO and WT cells and observed a 30% and 75% increase respectively (Fig 4D). Ki67 labelling confirmed the increased proliferation rate in KO cells, which was also validated by QPCR of Ki67 mRNA (Fig 4E). A TUNEL assay revealed no difference in the rate of apoptosis between mutant and control cells (Fig 4E).

To ascertain the role of OCAM in the observed effects, we constructed 2 lentiviruses to express the TM and GPI forms of the OCAM protein. The KO and wild-type cells were transduced and cytometric analysis showed that over 80% of KO cells re-expressed OCAM after infection (Fig 4F). We also confirmed the re-expression of OCAM in KO cells by WB however at a level lower than in wild-type cells (Fig 4B). Growth assays presented on Fig 4G indicated that, compared to control virus, rescuing the TM- or GPI forms of OCAM in KO cells decreased the number of cells obtained after 5 days of cultures. In addition, the ability to form new neurospheres at clonal density was reduced after re-expression of the TM form but, surprisingly not with the GPI form (Fig 4H). Overexpression of OCAM in WT cells also negatively affected the growth of these cells and their ability to form new neurospheres (Fig 4G and 1H).

Finally, as OCAM could also be found secreted in the media, we examined the effect of directly adding purified recombinant OCAM-Fc protein on the growth of wild-type cells. Fig 4I shows that the addition of OCAM in the medium reduced the cell number obtained after 7 days. Unexpectedly, this was also observed when OCAM KO cells were used suggesting that OCAM acts through heterophilic interactions.

## OCAM regulates ErbB2 in neural stem cells

**Fig 5:**
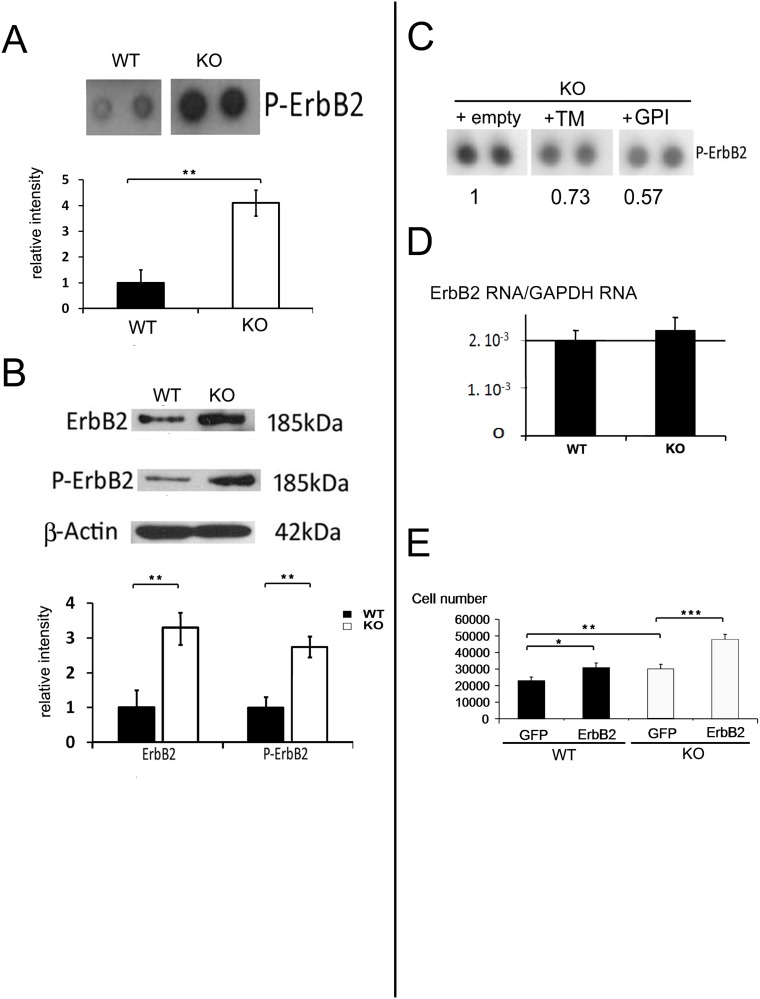
OCAM and ErbB2 signalling. (***A***): Detection of phosphorylated ErbB2 (P-ErbB2) by phospho-RTK array in WT and KO cultures. Bottom: blot quantification (n = 4). (***B***): Detection of ErbB2 and phospho-ErbB2 by WB in indicated cell extracts. Bottom: blot quantifications (n = 4). (***C***): phospho RTK array detection of phosphorylated ErbB2 protein in KO cultures transduced with empty (vect.), OCAM-TM or OCAM-GPI lentiviruses. Quantifications are indicated taking empty lentivirus transduced cells as a reference. This experiment is representative of 2 separate experiments. (***D***): QPCR quantification of ErbB2/GAPDH messenger ratio in indicated cells. No significant difference was observed (n = 4). (***E***): Influence of ErbB2 overexpression on neurosphere growth. KO and WT cultures were transfected with GFP- or ErbB2-expressing plasmids. Cell numbers were measured 7 days after seeding of transfected cells (n = 7).

ErbB receptors are a family of tyrosine kinase receptors, which bind cytokines of the EGF family [33]. These receptors are critically involved in the regulation of many cellular processes, notably in the control of proliferation and migration. Recent results in Drosophila showed that the Fas2 protein, a protein orthologous to OCAM and NCAM1, indirectly interacts with these receptors [28]. Drosophila mutant for Fas2 showed an enhanced activity for ErbB1 [28]. We thus investigated whether OCAM influenced the state of phosphorylation of ErbB receptors in E13 spinal cord neurospheres by using an antibody array for detection of phosphorylated tyrosine kinase receptors (phospho-RTK array) including ErbB receptors. Probing this array with proteins extracted from OCAM KO and wild-type neurospheres revealed a striking enhancement of phosphorylation of the ErbB2 receptor (Fig 5A) in KO cells. An antibody recognizing all forms of the ErbB2 receptor and an antibody recognizing the phosphorylated form were used to confirm this result by Western blot analysis. Fig 5B shows that the total quantity of ErbB2 was increased in KO cells as well as its phosphorylated form. To explore further the relationships between OCAM and ErbB2, we reintroduced the TM and GPI-linked forms of OCAM in KO cells by lentivirus transduction and then measured the phosphorylation of ErbB2 using a phospho-RTK array. Fig 5C shows that both isoforms were able to reduce ErbB2 phosphorylation.

The elevated quantity of ErbB2 protein observed in KO cells could be due to an increase in RNA level. To test this possibility, a QPCR analysis was carried out to quantify ErbB2 transcripts in KO and wild-type cells, but no significant difference was observed (Fig 5D). Finally, to gain further insight into the role of ErbB2 in regulating the growth of embryonic neurospheres, we analysed the effect of overexpressing ErbB2 in OCAM KO and WT cells. As indicated in Fig 5E, seven days after transfection and compared to control plasmid, ErbB2 was able to significantly increase the number of WT and mutant cells

## Discussion

Previously, OCAM has mainly been studied in the context of olfaction where it is involved in the formation or maintenance of dendritic and axonal compartmentalization in the olfactory glomeruli [29]. Here we report on OCAM in the context of spinal cord both in vitro and in vivo. OCAM was found strongly expressed in embryonic and adult neural stem cell cultures derived from the spinal cord. This adhesion protein was present at the surface of the majority of the cells including the small neurosphere-forming cell subpopulation. In vivo, OCAM was also detected during spinal cord development where it was detected in subpopulations of Map2^+^ neuronal cells and Sox2^+^ ependymal cells. Additionally, we demonstrated that OCAM is also present, albeit at a low level, in adult ependymal cells around the spinal cord central canal.

Not only is OCAM detected in neural stem cells but it is also modulated during their differentiation and activation. In adult, Sox2^+^ ependymal cells constitute a dormant stem cell niche which is rapidly activated after spinal cord injury. In this situation, these cells proliferate and migrate to the lesion site to participate to the glial scar [30]. During activation of the niche, OCAM was markedly increased at the RNA and protein level, suggesting a role for this protein in the activation process. The expression of OCAM is also regulated during neural stem cell differentiation. Indeed, OCAM expression is lost during differentiation of these cells into astrocytes while it remains expressed in neuronal and oligodendrocyte cells. The differentiation process is also accompanied by an increase in the ratio of GPI vs TM forms of OCAM. The reasons behind these complex regulations of OCAM are unclear and need further clarification. Using embryonic neurospheres KO for OCAM, we did not detect an influence of OCAM in regulating the proportion of neuronal versus glial cells obtained after differentiation.

Contrasting with this lack of effect in differentiation, we observed that the loss of OCAM increased neurosphere cell growth, which was associated with an increase in Ki67 expression together with a higher clonogenic property. It could appear contradictory that on the one hand OCAM^-^ cells purified from OCAM WT cultures do not form neurospheres (Fig 1G) and that on the other hand OCAM KO cultures, which are also OCAM^-^, have an increased proliferation and self-renewal rates (Fig 4). In fact, after neurosphere differentiation, astrocytic cells are OCAM^-^ cells (Fig 1H) and it is likely that the small percent of OCAM^-^ cells found in growing neurospheres are differentiated cells which are present at a low level even in undifferentiated cultures (our own observation). These OCAM^-^ cells are different from OCAM^-^ cells obtained from OCAM KO animal. In this case, the vast majority of OCAM^-^ cells in neurospheres represent undifferentiated growing cells which are still able neurospheres.

Cell adhesion molecules are now known to mediate much more than adhesion [31]. Indeed, these proteins participate actively in several canonical signalling pathways such as tyrosine kinase receptor signalling for instance and some of them (NCAM1) can also act as receptor for cytokines (GDNF) [32]. This implication of OCAM in the control of spinal cord neurosphere proliferation is reminiscent of the observed role of NCAM1 and L1CAM in brain neural stem cells. Addition of NCAM1 to hippocampal progenitors reduced their proliferation and enhanced their differentiation toward the neuronal lineage [5,7]. Likewise, upon contact with L1CAM, neural precursor cells showed a reduced proliferation and an increased neuronal differentiation [6]. Importantly, these effects are obtained through binding of NCAM1 and L1CAM to heterophilic partners. Likewise, we observed that addition of soluble OCAM on OCAM KO cells led to a reduced cell growth thus demonstrating that at least part of the OCAM effect is mediated by heterophilic interactions with an unknown partner.

Exploration of the molecular mechanisms involved in the overgrowth of mutant cells led us to uncover an unexpected link between OCAM and the ErbB2 protein. The ErbB/HER receptor family, which includes 4 members (EGFR/ErbB1, ErbB2, ErbB3, ErbB4), are transmembrane growth factor receptors that function to activate intracellular signalling pathways in response to extracellular signals [33]. These proteins are central for many cellular processes especially proliferation and migration. ErbB2 has oncogenic properties and is found overexpressed in up to 25% of human breast cancers where it is predictive of poor prognosis [34]. Unlike other members of the ErbB family, ErbB2 was long thought to lack ligand-binding activity and its signalling function was considered as strictly dependent on the formation of heterodimeric ErbB proteins. However, more recent data indicate that ErbB2 physically interacts with β4 integrin and also with the membrane mucin Muc4 [35]. In OCAM KO neurospheres, both the total level of ErbB2 and its phosphorylated forms were increased. The mRNA level was almost identical in KO and WT cells thus excluding a transcriptional effect of OCAM on ErbB2. This upregulation of ErbB2 in OCAM KO cells is likely to be involved in the growth increase as we observed that the overexpression of ErbB2 in WT neurospheres had a similar effect. In addition, reintroduction of OCAM in KO cells both reduced the cell growth and the level of phosphorylated ErbB2, thus reinforcing the functional links between these proteins and the neurosphere growth. Evidence for a regulation of the ErbB signalling by CAMs, as illustrated here, has also been observed in other contexts. Using a genetic screen to identify regulators for the EGF receptor in Drosophila, Mao et al, identified the Fasciclin 2 protein (Fas2), the NCAM1 ortholog in Drosophila, as an inhibitor of EGFR signalling activity during development [28]. In addition, in mammalian cells, it was shown that overexpression of NCAM1 reduced the basal level of EGFR [36] and enhanced the EGF-induced receptor down-regulation. In the spinal cord neural stem cell context, the mechanism by which the loss of OCAM increases ErbB2 level remains to be fully explored. Using immunoprecipitation, we have not been able to demonstrate a direct interaction between OCAM and ErbB2 and no clear co-localization of the two proteins was observed using confocal microscopy in neurosphere cells (data not shown). Thus it appears improbable that OCAM regulates ErbB2 by direct physical interactions as observed for instance between L1CAM and EGFR [37]. It has been shown that NCAM1 can indirectly regulate the ubiquitin-mediated degradation of EGFR through phosphorylation of the ubiquitin ligase c-Cbl [36]. Likewise, OCAM might influence the degradation of ErbB2 by its cognate ubiquitin ligases, notably CHIP [38] and c-Cbl ligase [39].

Using electron microscopy and an internalization assay, we observed that OCAM localization is dynamic and that in addition to the membrane, OCAM was also associated with a pool of internal vesicles. The expression and activation of ErbB family receptors are highly-regulated by endocytic recycling or lysosomial degradation which is based on a complex machinery involving vesicle trafficking [40]. A role for OCAM in controlling ErbB2 trafficking and degradation can thus be hypothesized, especially as a functional role in vesicle trafficking was documented for the OCAM-related protein NCAM1 in adrenal chromaffin cells [41]. Binding of NCAM1 to FGFR1 was also shown to control the intracellular trafficking of the receptor [42].

In conclusion, this study uncovered an unanticipated expression of the OCAM protein in spinal cord neural stem cells cultured as neurospheres and described its implication in the regulation of cell proliferation through the ErbB2 protein. This study provides evidence supporting the central role of cell adhesion molecules in the regulation of ErbB signalling.

## Supporting Information

S1 Fig Original detection of OCAM expression in embryonic spinal cord neurosphere cells. (***A***): Gene arrays specific for cytokines and adhesion molecules (purchased from R&D) were probed with cDNAs derived from growing (red) and differentiated (green) neurospheres as described in [12]. OCAM as well as VCAM1 mRNA were readily detected in both culture conditions. (***B***): High magnifications of OCAM spots.
(TIF)

S2 Fig OCAM expression during spinal cord development and in the adult central canal stem cell niche. (***A***): Expression of OCAM detected by immunofluorescence in the embryonic spinal cord. (***B***): High magnification of OCAM staining at E13.5. V = ventricle. (***C***): Immunodetection of Sox2 in E13.5 spinal cord. V = ventricle. ***C’*, *C”***: Examples of double positive Sox2^+^ OCAM^+^ cells in the ventricular zone. (***D***): Immunodetection of Map2 in E13.5 spinal cord. ***D’*, *D”***: Examples of double positive Map2^+^ OCAM^+^ cells in the mantle zone. (***E***): Immunodetection of OCAM in ependymal cells surrounding the central canal in adult mice. *Left*: OCAM is weakly expressed in the dormant niche. *Right*: 72h after spinal cord injury, OCAM is readily detected. Left and right images were taken with the same exposure time. (***F***): Detection of OCAM RNA by microarray analysis (top) (n = 4) or QPCR (bottom) (n = 4). Scale bars = 10 μm.
(TIF)

## References

[1] Hugnot JP, Franzen R. The spinal cord ependymal region: a stem cell niche in the caudal central nervous system. Front Biosci (Landmark Ed). 2011;16: 1044–1059. 21196217 10.2741/3734

[2] Sabelstrom H, Stenudd M, Frisen J. Neural stem cells in the adult spinal cord. Exp Neurol. 2014;260: 44–49. 10.1016/j.expneurol.2013.01.026 23376590

[3] Louis SA, Rietze RL, Deleyrolle L, Wagey RE, Thomas TE, Eaves AC, et al Enumeration of neural stem and progenitor cells in the neural colony-forming cell assay. Stem Cells. 2008;26: 988–996. 10.1634/stemcells.2007-0867 18218818

[4] Bian S. Cell Adhesion Molecules in Neural Stem Cell and Stem Cell- Based Therapy for Neural Disorders In: Bonfanti DL, editor. Neural Stem Cells—New Perspectives,: InTech; 2013.

[5] Amoureux MC, Cunningham BA, Edelman GM, Crossin KL. N-CAM binding inhibits the proliferation of hippocampal progenitor cells and promotes their differentiation to a neuronal phenotype. J Neurosci. 2000;20: 3631–3640. 10804205 10.1523/JNEUROSCI.20-10-03631.2000 PMC6772667

[6] Dihne M, Bernreuther C, Sibbe M, Paulus W, Schachner M. A new role for the cell adhesion molecule L1 in neural precursor cell proliferation, differentiation, and transmitter-specific subtype generation. J Neurosci. 2003;23: 6638–6650. 12878705 10.1523/JNEUROSCI.23-16-06638.2003 PMC6740621

[7] Shin MH, Lee EG, Lee SH, Lee YS, Son H. Neural cell adhesion molecule (NCAM) promotes the differentiation of hippocampal precursor cells to a neuronal lineage, especially to a glutamatergic neural cell type. Exp Mol Med. 2002;34: 401–410. 12526081 10.1038/emm.2002.57

[8] Karpowicz P, Willaime-Morawek S, Balenci L, DeVeale B, Inoue T, van der Kooy D. E-Cadherin regulates neural stem cell self-renewal. J Neurosci. 2009;29: 3885–3896. 10.1523/JNEUROSCI.0037-09.2009 19321785 PMC6665048

[9] Noles SR, Chenn A. Cadherin inhibition of beta-catenin signaling regulates the proliferation and differentiation of neural precursor cells. Mol Cell Neurosci. 2007;35: 549–558. 17553695 10.1016/j.mcn.2007.04.012

[10] Zhang J, Woodhead GJ, Swaminathan SK, Noles SR, McQuinn ER, Pisarek AJ, et al Cortical neural precursors inhibit their own differentiation via N-cadherin maintenance of beta-catenin signaling. Dev Cell. 2010;18: 472–479. 10.1016/j.devcel.2009.12.025 20230753 PMC2865854

[11] Sabourin JC, Ackema KB, Ohayon D, Guichet PO, Perrin FE, Garces A, et al A mesenchymal-like ZEB1(+) niche harbors dorsal radial glial fibrillary acidic protein-positive stem cells in the spinal cord. Stem Cells. 2009;27: 2722–2733. 10.1002/stem.226 19785035

[12] Deleyrolle L, Marchal-Victorion S, Dromard C, Fritz V, Saunier M, Sabourin JC, et al Exogenous and fibroblast growth factor 2/epidermal growth factor-regulated endogenous cytokines regulate neural precursor cell growth and differentiation. Stem Cells. 2006;24: 748–762. 16166253 10.1634/stemcells.2005-0138

[13] Yoshihara Y, Kawasaki M, Tamada A, Fujita H, Hayashi H, Kagamiyama H, et al OCAM: A new member of the neural cell adhesion molecule family related to zone-to-zone projection of olfactory and vomeronasal axons. J Neurosci. 1997;17: 5830–5842. 9221781 10.1523/JNEUROSCI.17-15-05830.1997 PMC6573213

[14] Walz A, Mombaerts P, Greer CA, Treloar HB. Disrupted compartmental organization of axons and dendrites within olfactory glomeruli of mice deficient in the olfactory cell adhesion molecule, OCAM. Mol Cell Neurosci. 2006;32: 1–14. 16531066 10.1016/j.mcn.2006.01.013

[15] Alenius M, Bohm S. Differential function of RNCAM isoforms in precise target selection of olfactory sensory neurons. Development. 2003;130: 917–927. 12538518 10.1242/dev.00317

[16] Borisovska M, McGinley MJ, Bensen A, Westbrook GL. Loss of olfactory cell adhesion molecule reduces the synchrony of mitral cell activity in olfactory glomeruli. J Physiol. 2011;589: 1927–1941. 10.1113/jphysiol.2011.206276 21486802 PMC3090595

[17] Schmitt-Ulms G, Hansen K, Liu J, Cowdrey C, Yang J, DeArmond SJ, et al Time-controlled transcardiac perfusion cross-linking for the study of protein interactions in complex tissues. Nat Biotechnol. 2004;22: 724–731. 15146195 10.1038/nbt969

[18] Nakai S, Kawano H, Yudate T, Nishi M, Kuno J, Nagata A, et al The POU domain transcription factor Brn-2 is required for the determination of specific neuronal lineages in the hypothalamus of the mouse. Genes Dev. 1995;9: 3109–3121. 8543155 10.1101/gad.9.24.3109

[19] Callahan CA, Thomas JB. Tau-beta-galactosidase, an axon-targeted fusion protein. Proc Natl Acad Sci U.S.A. 1994;91: 5972–5976. 8016099 10.1073/pnas.91.13.5972 PMC44119

[20] Minowa O, Ikeda K, Sugitani Y, Oshima T, Nakai S, Katori Y, et al Altered cochlear fibrocytes in a mouse model of DFN3 nonsyndromic deafness. Science. 1999;285: 1408–1411. 10464101 10.1126/science.285.5432.1408

[21] Yagi T, Ikawa Y, Yoshida K, Shigetani Y, Takeda N, Mabuchi I, et al Homologous recombination at c-fyn locus of mouse embryonic stem cells with use of diphtheria toxin A-fragment gene in negative selection. Proc Natl Acad Sci U.S.A. 1990;87: 9918–9922. 2263643 10.1073/pnas.87.24.9918 PMC55285

[22] Sakai K, Miyazaki J. A transgenic mouse line that retains Cre recombinase activity in mature oocytes irrespective of the cre transgene transmission. Biochem Biophys Res Commun. 1997;237: 318–324. 9268708 10.1006/bbrc.1997.7111

[23] Salmon P, Trono D. Production and titration of lentiviral vectors Curr protoc Hum Genet. In: Jonathan L, Haines JL editors. 2007;Chapter 12:Unit 12.10. 10.1002/0471142905.hg0808s52 18428406

[24] Hartfuss E, Galli R, Heins N, Gotz M. Characterization of CNS precursor subtypes and radial glia. Dev Biol. 2001;229: 15–30. 11133151 10.1006/dbio.2000.9962

[25] Slezak M, Goritz C, Niemiec A, Frisen J, Chambon P, Metzger D, et al Transgenic mice for conditional gene manipulation in astroglial cells. Glia. 2007;55: 1565–1576. 17823970 10.1002/glia.20570

[26] Mich JK, Signer RA, Nakada D, Pineda A, Burgess RJ, Vue TY, et al Prospective identification of functionally distinct stem cells and neurosphere-initiating cells in adult mouse forebrain. Elife. 2014;3: e02669 10.7554/eLife.02669 24843006 PMC4038845

[27] Secher T. Soluble NCAM. Adv Exp Med Biol. 2010;663: 227–242. 10.1007/978-1-4419-1170-4_15 20017026

[28] Mao Y, Freeman M. Fasciclin 2, the Drosophila orthologue of neural cell-adhesion molecule, inhibits EGF receptor signalling. Development. 2009;136: 473–481. 10.1242/dev.026054 19141676 PMC2687591

[29] Winther M, Berezin V, Walmod PS. NCAM2/OCAM/RNCAM: cell adhesion molecule with a role in neuronal compartmentalization. Int J Biochem Cell Biol. 2012;44: 441–446. 10.1016/j.biocel.2011.11.020 22155300

[30] Sabelstrom H, Stenudd M, Frisen J. Neural stem cells in the adult spinal cord. Exp Neurol. 2014;260: 44–49. 10.1016/j.expneurol.2013.01.026 23376590

[31] Gibson NJ. Cell adhesion molecules in context: CAM function depends on the neighborhood. Cell Adh Migr. 2011;5: 48–51. 20948304 10.4161/cam.5.1.13639 PMC3038097

[32] Paratcha G, Ledda F, Ibanez CF. The neural cell adhesion molecule NCAM is an alternative signaling receptor for GDNF family ligands. Cell. 2003;113: 867–879. 12837245 10.1016/s0092-8674(03)00435-5

[33] Bublil EM, Yarden Y. The EGF receptor family: spearheading a merger of signaling and therapeutics. Curr Opin Cell Biol. 2007;19: 124–134. 17314037 10.1016/j.ceb.2007.02.008

[34] Harari D, Yarden Y. Molecular mechanisms underlying ErbB2/HER2 action in breast cancer. Oncogene. 2000;19: 6102–6114. 11156523 10.1038/sj.onc.1203973

[35] Carraway KL, Theodoropoulos G, Kozloski GA, Carothers Carraway CA. Muc4/MUC4 functions and regulation in cancer. Future Oncol. 2009;5: 1631–1640. 10.2217/fon.09.125 20001800 PMC2825673

[36] Povlsen GK, Berezin V, Bock E. Neural cell adhesion molecule-180-mediated homophilic binding induces epidermal growth factor receptor (EGFR) down-regulation and uncouples the inhibitory function of EGFR in neurite outgrowth. J Neurochem. 2008;104: 624–639. 17995934 10.1111/j.1471-4159.2007.05033.x

[37] Islam R, Kristiansen LV, Romani S, Garcia-Alonso L, Hortsch M. Activation of EGF receptor kinase by L1-mediated homophilic cell interactions. Mol Biol Cell. 2004;15: 2003–2012. 14718570 10.1091/mbc.E03-05-0333 PMC379294

[38] Xu W, Marcu M, Yuan X, Mimnaugh E, Patterson C, Neckers L. Chaperone-dependent E3 ubiquitin ligase CHIP mediates a degradative pathway for c-ErbB2/Neu. Proc Natl Acad Sci U.S.A. 2002;99: 12847–12852. 12239347 10.1073/pnas.202365899 PMC130548

[39] Klapper LN, Waterman H, Sela M, Yarden Y. Tumor-inhibitory antibodies to HER-2/ErbB-2 may act by recruiting c-Cbl and enhancing ubiquitination of HER-2. Cancer Res. 2000;60: 3384–3388. 10910043

[40] Sorkin A, Goh LK. Endocytosis and intracellular trafficking of ErbBs. Exp Cell Res. 2009;315: 683–696. 19278030 10.1016/j.yexcr.2008.07.029 PMC2605728

[41] Chan SA, Polo-Parada L, Landmesser LT, Smith C. Adrenal chromaffin cells exhibit impaired granule trafficking in NCAM knockout mice. J Neurophysiol. 2005;94: 1037–1047. 15800072 10.1152/jn.01213.2004

[42] Francavilla C, Cattaneo P, Berezin V, Bock E, Ami D, de MArco A, et al The binding of NCAM to FGFR1 induces a specific cellular response mediated by receptor trafficking. J Cell Biol. 2009;187: 1101–1116. 10.1083/jcb.200903030 20038681 PMC2806277

