# Supplementary figures and images for: OCAM Regulates Embryonic Spinal Cord Stem Cell Proliferation by Modulating ErbB2 Receptor
Source: PLoS One. 2015 Apr 13;10(4):e0122337. doi: 10.1371/journal.pone.0122337 (PMC4395419; doi:10.1371/journal.pone.0122337)

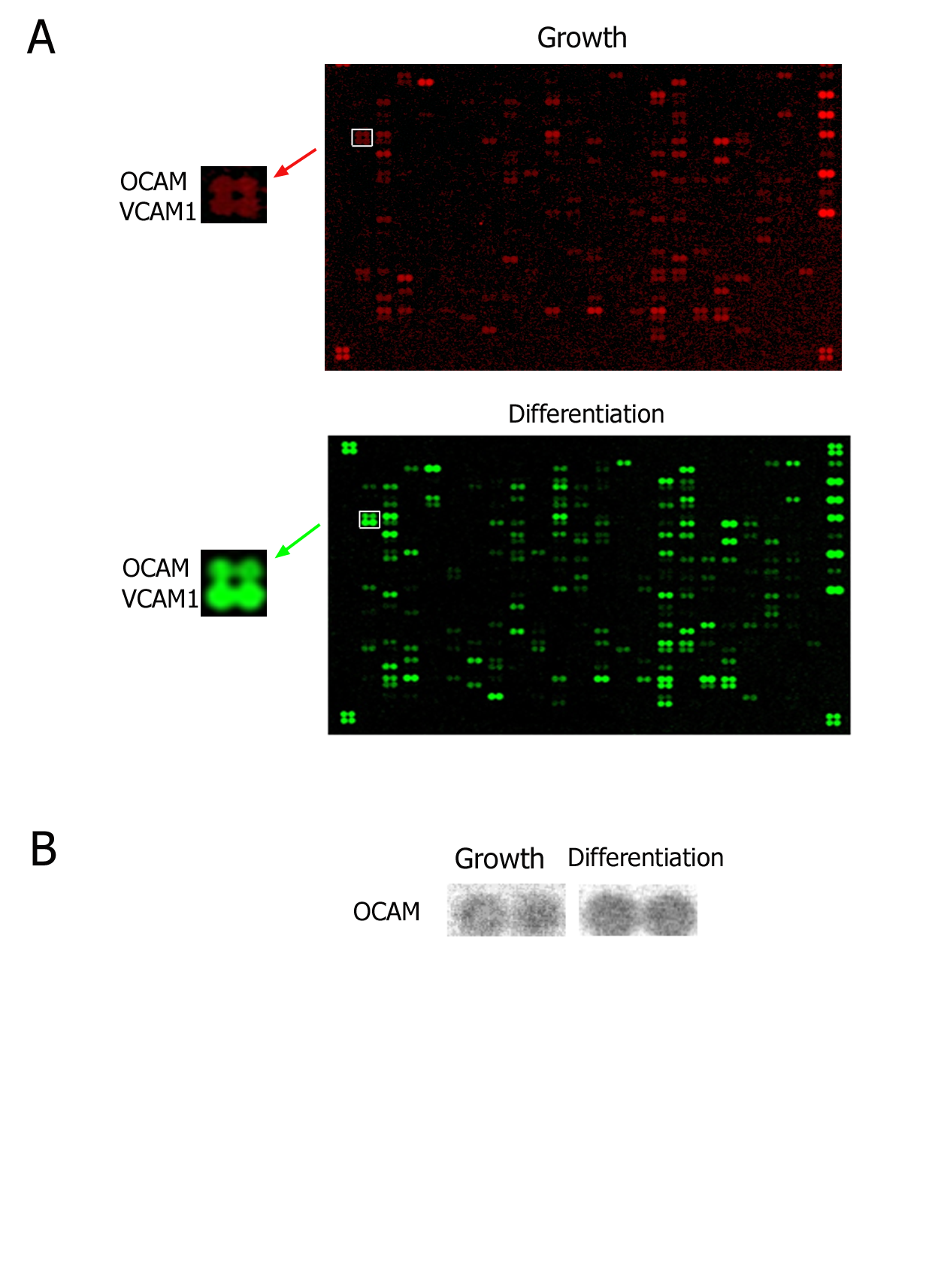

Supplement: S1 Fig — (A): Gene arrays specific for cytokines and adhesion molecules (purchased from R&D) were probed with cDNAs derived from growing (red) and differentiated (green) neurospheres as described in [12]. OCAM as well as VCAM1 mRNA were readily detected in both culture conditions. (B): High magnifications of OCAM spots. (TIF) [file pone.0122337.s001.tif]

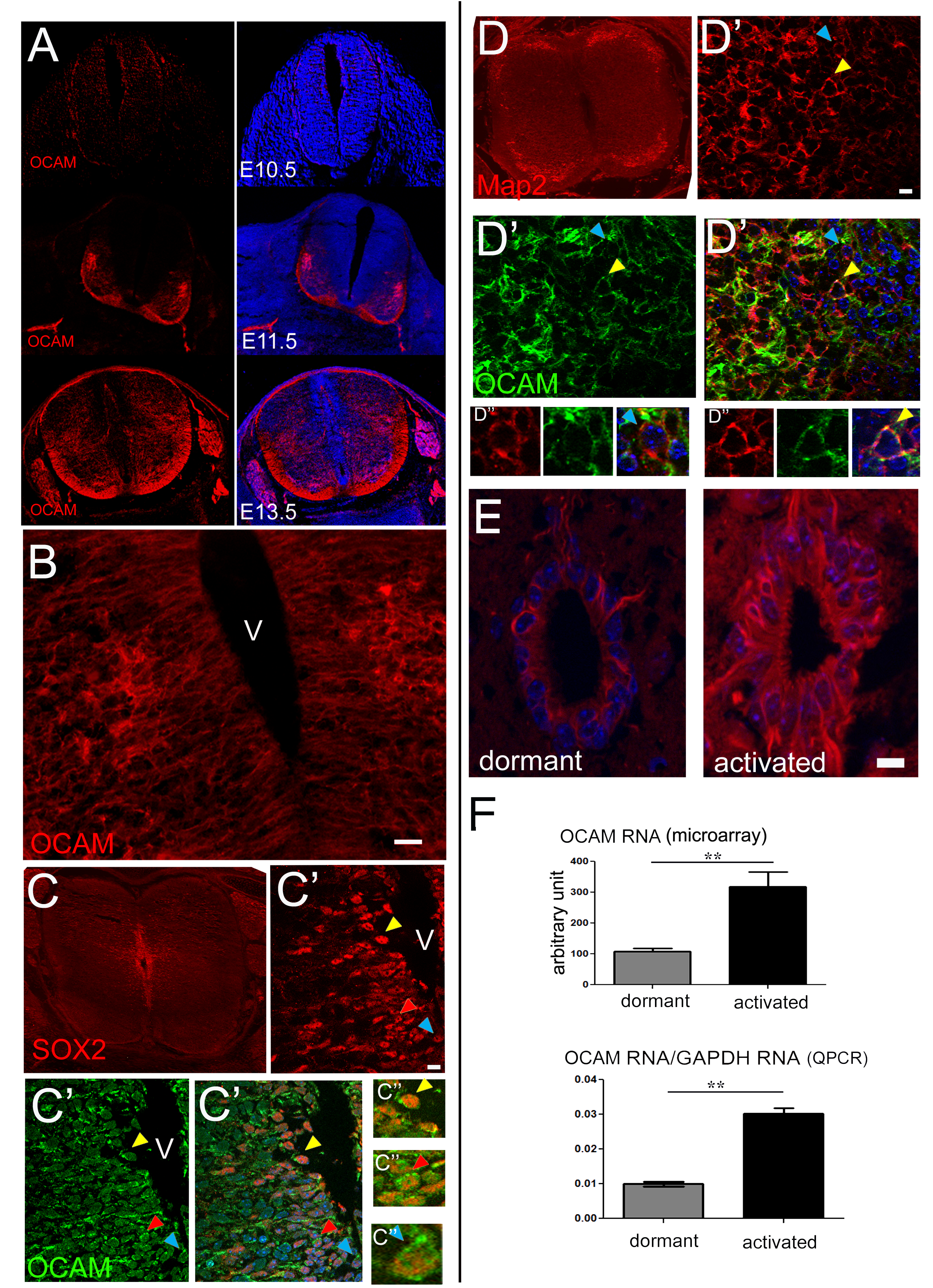

Supplement: S2 Fig — (A): Expression of OCAM detected by immunofluorescence in the embryonic spinal cord. (B): High magnification of OCAM staining at E13.5. V = ventricle. (C): Immunodetection of Sox2 in E13.5 spinal cord. V = ventricle. C’, C”: Examples of double positive Sox2+ OCAM+ cells in the ventricular zone. (D): Immunodetection of Map2 in E13.5 spinal cord. D’, D”: Examples of double positive Map2+ OCAM+ cells in the mantle zone. (E): Immunodetection of OCAM in ependymal cells surrounding the central canal in adult mice. Left: OCAM is weakly expressed in the dormant niche. Right: 72h after spinal cord injury, OCAM is readily detected. Left and right images were taken with the same exposure time. (F): Detection of OCAM RNA by microarray analysis (top) (n = 4) or QPCR (bottom) (n = 4). Scale bars = 10 μm. (TIF) [file pone.0122337.s002.tif]
